# Injuries due to fall make summer time power outages a potential public health issue

**DOI:** 10.4103/0974-2700.76817

**Published:** 2011

**Authors:** Muhammad S Shamim, Uzma R Khan, Junaid A Razzak, Jooma Rasheed

**Affiliations:** 1 Department of Surgery, Government of Pakistan, Pakistan; 2 Department of Emergency Medicine, Government of Pakistan, Pakistan; 3 Department of Aga Khan University Hospital, Karachi - 74800, Government of Pakistan, Pakistan; 4 Department of Health, Government of Pakistan, Pakistan

Sir,

Falls are a significant cause of morbidity and mortality, especially in children and in under-developed countries.[1–7] Unexpected power outages in Karachi have become a frequent problem. This study was carried out at Jinnah Postgraduate Medical Centre, Karachi, to explore an association between increasing power outages and frequency of falls. Data for all injuries were collected prospectively, and the year 2006 was chosen for analysis at it had worst power outages.[8,9] Injuries were classified as those occurring in summer (April to July) or winter (December to March) and were compared between the two seasons. Total injuries in study duration was 2599, mean age was 21 ± 18 years (male:female 3.2:1) and 43% of victims were children. Injuries in two seasons were comparable (summer 1266, winter 1333) but injuries due to fall were more in summer (574 versus 470, OR= 1.5; 95% CI = 1.3, 1.8), especially in children (*P* = 0.001). Frequency of falls were much more in 2006 as compared with either 2005 (OR = 1.59; 95% CI = 1.37, 1.83) or 2004 (OR = 1.22; 95% CI = 1.05, 1.42).

Karachi’s hot and humid climate and frequent power outages encourages families to spend time on rooftops or balconies, or to leave the windows open. Understandably, we found a higher frequency of falls, especially of children in all summer months, observations shown by others as well.[3,5,6] However, to the best of our knowledge, this is the first study which points toward a possible association between increasing power outages and falls, especially involving children, implying power outages as a potential health hazard. On a similar note but in an area with cold climate, investigators have previously found an association between power outages and heater related injuries.[10] Larger, more in depth, prospective studies are recommended to validate our findings.
